# Supplementary figures and images for: Enhancing the Therapeutic Effect and Bioavailability of Irradiated Silver Nanoparticle-Capped Chitosan-Coated Rosuvastatin Calcium Nanovesicles for the Treatment of Liver Cancer
Source: Pharmaceutics. 2025 Jan 7;17(1):72. doi: 10.3390/pharmaceutics17010072 (PMC11769262; doi:10.3390/pharmaceutics17010072)

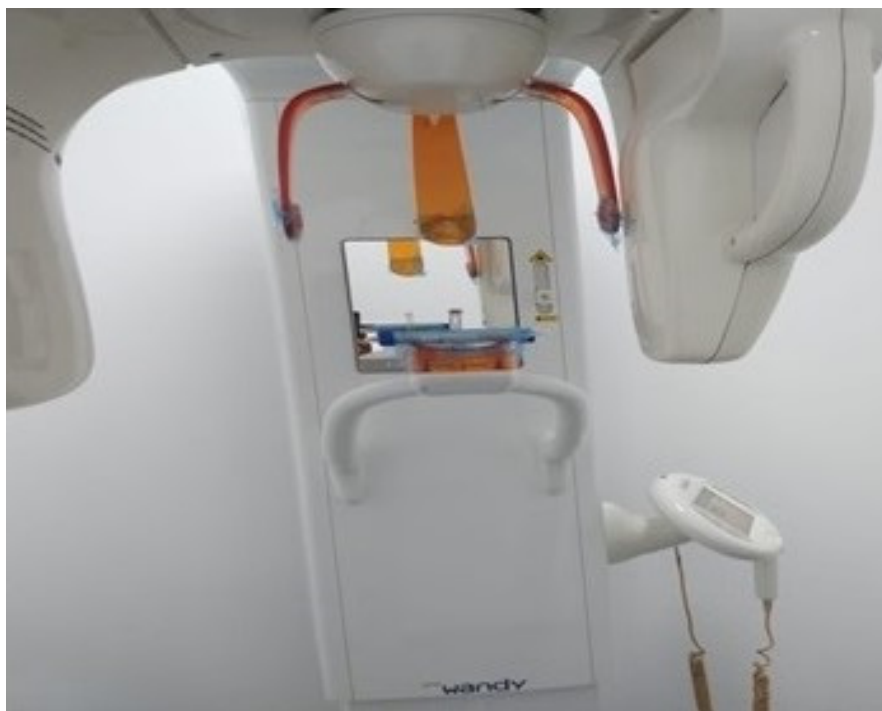

**Figure S1** Exposure of tested formulations to X-ray

Supplement: Supplementary file 1 [file pharmaceutics-17-00072-s001.zip › pharmaceutics-3350199 supplementary.pdf]
